# Association of epigenetic inactivation of the *WRN* gene with anticancer drug sensitivity in cervical cancer cells

**DOI:** 10.3892/or.2012.1912

**Published:** 2012-07-13

**Authors:** KENTA MASUDA, KOUJI BANNO, MEGUMI YANOKURA, KOSUKE TSUJI, YUSUKE KOBAYASHI, IORI KISU, ARISA UEKI, WATARU YAMAGAMI, HIROYUKI NOMURA, EIICHIRO TOMINAGA, NOBUYUKI SUSUMU, DAISUKE AOKI

**Affiliations:** Department of Obstetrics and Gynecology, School of Medicine, Keio University, Tokyo, Japan

**Keywords:** *WRN*, cervical cancer, DNA hypermethylation, CPT-11, chemosensitivity

## Abstract

The Werner (*WRN*) gene codes for a DNA helicase that contributes to genomic stability and has been identified as the gene responsible for progeria. Recent studies have shown reduced *WRN* expression due to aberrant DNA hypermethylation in cancer cells. Furthermore, *WRN* expression is thought to affect sensitivity to DNA topoisomerase I inhibitors in cancer therapy. In this study, we examined the relationship between aberrant DNA hypermethylation of *WRN* and the sensitivity of cervical cancer cells to anticancer drugs. DNA was extracted from samples from 22 patients with primary cervical cancer and 6 human cervical cancer-derived cell lines. Aberrant DNA hypermethylation was analyzed by methylation-specific PCR. *WRN* expression in cultured cells before and after addition of 5-aza-2-deoxycytidine, a demethylating agent, was examined using RT-PCR. The sensitivity of cells to anticancer drugs was determined using a collagen gel droplet embedded culture drug sensitivity test (CD-DST). siRNA against *WRN* was transfected into a cervical cancer-derived cell line with high *WRN* expression. Changes in drug sensitivity after silencing *WRN* were determined by CD-DST. Aberrant DNA hypermethylation and decreased expression of *WRN* were detected in 7/21 cases of primary cervical cancer and in two cervical cancer-derived cell lines. These two cell lines showed high sensitivity to CPT-11, a topoisomerase I inhibitor, but became resistant to CPT-11 after treatment with 5-aza-2-deoxycytidine. Transfection of siRNA against *WRN* increased the sensitivity of the cells to CPT-11. Aberrant DNA hypermethylation of *WRN* also increased the sensitivity of cervical cancer cells to CPT-11. Therefore, epigenetic inactivation of this gene may be a biomarker for selection of drugs for the treatment of cervical cancer. This is the first report to show a relationship between the methylation of the *WRN* gene and sensitivity to CPT-11 in gynecological cancers.

## Introduction

The incidence of cervical cancer has decreased in advanced countries due to more frequent health checkups and the development of vaccines. In the United States in 2010, there were 12,200 cases of cervical cancer and 4,210 deaths related to this disease, reflecting a decreasing tendency (1). However, cases of cervical cancer worldwide have increased from 378,000 in 1980 to 454,000 in 2010 (2). Death due to cervical cancer has decreased, but there were still about 200,000 deaths worldwide in 2010, including 46,000 females aged 15–49 years in developing countries (2).

Cervical cancer occurs due to infection with the human papillomavirus (HPV) (3). More than 100 genotypes of HPV have been detected and about 40 have been found to infect the genital tract. HPV is classified into high-risk types causing cervical cancer, including HPV16 and HPV18, and low-risk types causing conditions other than cancer, such as a polyp. HPV16, the most frequent genotype, is detected in approximately half of patients with cervical cancer (4). After infecting cells, HPV produces oncoproteins E6 and E7, which inhibit controlling the cell cycle and apoptosis, and therefore have important roles in oncogenesis. E6 is associated with p53 and induces p53 degradation by E3 ubiquitin ligase via the function of E6-associated protein (E6AP). E7 inactivates the retinoblastoma tumor suppressor gene product, pRb and its family members (5).

Epigenetic DNA methylation of tumor suppressor genes in the promoter region is generally important in carcinogenesis (6–8). In cervical cancer, carcinogenesis is related to aberrant methylation of CpG islands of p16, FHIT, retinoic acid receptor β, E-cadherin, death-associated protein kinase, HIC-1, APC, and Ras association domain family 1A genes (9–12). Methylation of CpG islands in the *WRN* promoter region is related to carcinogenesis in various cancers (13). The chromosomal *WRN* locus on the short arm of chromosome 8, is composed of 35 exons, and has a length >250 kb (14). *WRN* encodes the *WRN* protein, which is a member of the RecQ helicase family and is also an exonuclease. Loss of *WRN* causes abnormalities in DNA repair, replication, and telomere maintenance.

Werner syndrome (WS) is an autosomal recessive genetic disease that is caused by mutation of the *WRN* gene. WS symptoms include aging at an early stage and various secondary symptoms associated with aging, including bilateral cataract, skin change, short stature and graying hair; in addition, diabetes mellitus, osteoporosis, atherosclerosis and cancer also develop frequently (15). Malignant complications include sarcomas of mesenchymal origins, including soft-tissue sarcoma and osteosarcoma, suggesting that the mechanism of carcinogenesis in WS differs from that in carcinogenesis in other cancers (16). The mean age at death of WS patients is 46–54 years and one of major causes is the high prevalence of malignancy (17,18).

Several studies have shown a relationship between *WRN* expression and malignancy and have indicated that epigenetic inactivation of *WRN* is of importance in carcinogenesis. In many tumors, loss of heterozygosity is detected in chromosome 8p, in which *WRN* is located, but *WRN* somatic mutation has not been found, suggesting that epigenetic control has a significant effect (19,20). Epigenetic DNA methylation in the promoter region of *WRN* and a methylation-induced decrease in *WRN* expression have been found in colorectal, lung, gastric, prostate and breast cancer. The methylation-induced decrease in *WRN* expression increases chromosomal instability (13).

Reduced *WRN* expression is also related to sensitivity to camptothecin (CPT-11), a topoisomerase I (Top-I) inhibitor (13,21). CPT-11 is an alkaloid found in plants such as *Camptotheca acuminata*. A single-strand break (SSB) occurs after Top I binds to DNA and generates a Top I-DNA cleavable complex. CPT-11 stabilizes this complex and inhibits reconnection of the SSB, resulting in inhibition of DNA synthesis (22). CPT-11 also inhibits replication fork progression, resulting in DNA double-strand breaks (DSBs) and apoptosis (23). Inactivation of *WRN* in cancer cells increases the effect of CPT-11 (13,21), and overall survival of patients with colorectal cancer treated with irinotecan, a camptothecin analogue, is dependent on the methylation status of CpG islands in the *WRN* promoter (13).

Cisplatin is a key drug in chemotherapy for cervical cancer (24). CPT-11 is a similarly important drug and has a high response rate of 24% (25). Evaluation of *WRN* expression as a marker of sensitivity to CPT-11 may be clinically useful in treatment of cervical cancer. Thus, in this study, the associations among cervical cancer, *WRN* expression, and cancer cell sensitivity to CPT-11 were investigated.

## Materials and methods

### Subjects and cytologic specimens

Samples were obtained from 21 cervical cancer smears collected using a ThinPrep system (Cytyc, Boxborough, MA, USA) and kept in preservation fluid (PreservCyt Solution, Cytyc) (26). Informed consent was obtained before collection. Pathological diagnosis was performed by cervical histology, and the cytological and histological results were consistent for all smears. Of the 21 cervical cancer smears, 10 were squamous carcinoma and 11 were adenocarcinoma. The histological type and stage were determined according to the General Rules for Clinical Cervical Cancer in Japan published by the Japan Society of Obstetrics and Gynecology.

### Cultured cell lines

The human cervical squamous cell carcinoma-derived cell lines, SKG-I, SKG-II, SKG-IIIa and SKG-IIIb, and the human cervical adenocarcinoma-derived cell lines, HeLa and TCO-I, were used in the study. HeLa cells were incubated in DMEM (Sigma, St. Louis, MO, USA) with 10% fetal bovine serum (FBS) (Sanko Junyaku Co., Tokyo, Japan) and TCO-I cells were incubated in MEM medium (Sigma) with 10% FBS. All other cell lines were incubated in F12 medium (Sigma) with 10% FBS. Cells were incubated in 10-cm dishes at 37°C in a 5% CO_2_ atmosphere.

### DNA extraction and methylation-specific PCR (MSP) assay of the WRN gene

DNA was extracted from 21 cervical smears and 6 cervical carcinoma-derived cell lines using a Get Pure DNA kit (Dojin Glocal, Kumamoto, Japan). DNA (1 μg) extracted from cervical smears was diluted with 50 μl of distilled water and incubated in 5.5 μl of 3 N NaOH at 37°C for 15 min. To this solution, 30 μl of 10 mM hydroquinone (Sigma) and 520 μl of 3 M sodium bisulfite (prepared at pH 5.5 with 10 N NaOH, Sigma) were added with mixing. Mineral oil was laid over the solution to prevent evaporation, and the solution was incubated overnight at 50°C. The lower layer of the reaction solution was mixed with 1 ml of Clean-up Resin (Promega, Madison, WI, USA) and then injected into a column. After rinsing with 2 ml of 80% isopropanol, the mixture was centrifuged at 15,000 rpm for 3 min to remove isopropanol. Hot (70°C) distilled water (50 μl) was added and the mixture was centrifuged at 15,000 rpm for 2 min to elute DNA. The DNA was then incubated with 5.5 μl of 2 N NaOH at 37°C for 20 min. Next, 66 μl of 5 N ammonium acetate and 243 μl of 95% ethanol were added and the mixture was incubated at −80°C for one hour and centrifuged at 15,000 rpm for 30 min to precipitate DNA. Supernatant exceeding 50 μl was removed, 1 ml of 60% ethanol was added, and the mixture was centrifuged at 15,000 rpm for 30 min and rinsed. The precipitated DNA was dried in air and dissolved in 20 μl of distilled water. DNA solution (2 μl) was used as the MSP template. In the PCR assay, AmpliTaq Gold and 10X PCR buffer/MgCl_2_ with dNTP (Applied Biosystems, Foster City, CA, USA) were used and the results were analyzed with a GeneAmp PCR System 9700 (Applied Biosystems).

The primer sequences were 5′-CGGGTAGGGGTATCG TTCGC-3′ (sense) and 5′-AACGAAATCCACCGCCCGCC-3′ (antisense), 159 bp. Primer sequences for the unmethylated reaction were 5′-GTAGTTGGGTAGGGGTATTGTTTGT-3′ (sense) and 5′-AAACAACCTCCACCACCCACCCC-3′ (antisense), 165 bp. PCR was performed for 35 cycles (95, 65 and 72°C for 30 sec, respectively). DNA extracted from the cultured cell lines was prepared similarly for use in MSP analysis of the *WRN* gene.

### RNA extraction and RT-PCR assay of WRN expression

Total RNA from 6 cervical cancer-derived cell lines was extracted using an RNeasy mini-kit (Qiagen, Valencia, CA, USA). cDNA was synthesized from 1 μg of total RNA using SuperScriptII Reverse Transcriptase (Invitrogen, Carlsbad, CA, USA). *WRN* expression was analyzed in an RT-PCR assay using 1 μl of first-strand cDNA as template. AmpliTaq Gold and 10X PCR buffer/MgCl_2_ with dNTP were used in the PCR assay, with analysis using a GeneAmp PCR System 9700 (Applied Biosystems). The primer sequences were 5′-GCATGTGTTCGGAAGAGTGTTT-3′ (sense) and 5′-TGACATGGAAGAAACGTGGAA-3′ (antisense), 258 bp. PCR was performed for 30 cycles (94, 57 and 72°C for 30 sec, respectively).

### Demethylation treatment

Cervical carcinoma-derived SKGII and TCO-I cells with aberrant methylation of *WRN* were plated on 10-cm dishes at 10^6^ cell/dish and incubated for 72 h. 5-Aza-2-deoxycytidine (5-aza) (Sigma), a demethylating agent, was then added at a final concentration of 1 μM in culture medium. After 48 h of incubation, 5-aza was added again and DNA and RNA were extracted 48 h after the second addition of 5-aza.

### In vitro test of sensitivity to anticancer agents

The sensitivity of cervical carcinoma-derived cell lines to anti-cancer agents was determined using the collagen gel droplet-embedded culture drug sensitivity test (CD-DST) (27). Cells were pretreated with cell dispersion enzyme EZ (Nitta Gelatin Inc., Tokyo, Japan) for 2 h, followed by centrifugation to collect the cells. In a flask containing collagen gel, the cells were pre-incubated for 24 h and surviving cells that adhered to collagen gel were collected. Cellmatrix Type CD solution was added to the collected cells, and the suspension of cells and collagen gel was dropped onto a 6-well plate to prepare 3 drops of 30 μl each. The suspension was left to stand in an incubator at 37°C in a 5% CO_2_ atmosphere for 1 h for gelling and then overlaid with 4 ml/well of medium. Cisplatin (CDDP), doxorubicin (ADM), or CPT-11 was then added to the suspension at final concentrations of 0.2, 0.02 and 0.03 μg/ml, respectively. After 24 h, the anticancer agents were removed by rinsing and the cells were incubated without serum at 37°C in 5% CO_2_ for 7 days. The cells were dyed with neutral red, fixed with formalin, and dried. Images were collected by scanning using an image analyzer and the ratio of the volume of living cancer cells in the treated group (T) to that in the control group (C) (T/C ratio) was determined. In general, cells are considered to be highly sensitive to the agent when the T/C ratio is ≤50% (28).

### Transfection of small interfering RNA (siRNA)

SKG-IIIb cells were plated on 60-mm dishes at 4×10^5^ cell/dish and transfected 48 h later with siRNA using siFector (B-Bridge International Inc., Cupertino, CA, USA). In this procedure, 4.5 μl of siRNA stock solution (100 μM) and 295.5 μl of serum-free MEM were mixed in a test tube. In another tube, 13.5 μl of siFector and 286.5 μl of serum-free MEM were mixed. The solutions from the two tubes were mixed and incubated at room temperature for 30 min. Each dish containing SKG-IIIb cells was rinsed twice with 2 ml of serum-free MEM and 2.4 ml of serum-free MEM was then added. The incubated siRNA mixture was added to the dish at 0.6 ml/dish and incubated at 37°C in 5% CO_2_ for 6 h. After incubation, 3 ml of MEM containing 20% serum was added to the dish. A negative control siRNA was used as designed by B-Bridge International Inc. The siRNA sequence corresponding to the *WRN* gene was 5′-GUUCUUGUCACGUCCUCUGdTdT-3′. The expression levels of mRNA and protein were determined 48 h after siRNA addition. Anticancer agents were added 48 h after siRNA addition and the sensitivity of the cells to each agent was analyzed using the CD-DST.

### Immunoblotting

siRNA-transfected SKG-IIIb cells were rinsed with PBS, trypsinized, and centrifuged at 15,000 rpm for 5 min at 4°C. Protein was extracted using a Mammalian Cell Extraction kit (BioVision Research Products, Mountain View, CA, USA). The sample (200 μg of protein) was mixed with sample buffer (Bio-Rad Laboratories, Hercules, CA, USA) containing the equivalent volume of 5% β-mercaptoethanol (Bio-Rad Laboratories) and the mixture was boiled for 5 min. After boiling, the mixture was electrophoresed on a 10% polyacrylamide gel and the proteins were transferred to nitrocellulose membranes (Bio-Rad Laboratories). The membranes were soaked in PBS containing 1% BSA and 0.1% Tween-20 and incubated at room temperature for 1 h for blocking. They were then reacted with anti-β-actin antibody (AC-74, Sigma-Aldrich, St. Louis, MO, USA; 5000-fold diluted) and anti-*WRN* antibody (4H12, Abcam, Cambridge, UK; 500-fold diluted) at 4°C overnight, followed by rinsing three times with PBS containing 0.1% Tween (PBS-T) for 10 min each. The anti-β-actin and anti-WRN antibodies were reacted with anti-mouse IgG antibody (PK-6102, Vector Laboratories, Burlingame, CA, USA) and anti-goat IgG antibody (BA-5000, Vector Laboratories; 250-fold diluted), respectively, at room temperature for 1 h. The membranes were rinsed with PBS-T three times and reacted with ABC complex (PK-6102, Vector Laboratories; pre-reacted at 4°C for 30 min) at room temperature for 1 h, then rinsed with PBS-T twice and PBS once, and visualized with DAB (Sigma).

### Cell cycle analysis using flow cytometry

The cell cycle was evaluated 96 h after siRNA addition. Cells were trypsinized and rinsed twice with PBS. Supernatant was separated from the cell pellets by centrifugation at 1,000 rpm for 5 min, and 450 μl of PBS was added to the pellets and the mixture was pipetted well. As the mixture was vortexed, 1 ml of cool 100% ethanol was added. The mixture was then incubated at room temperature for 30 min for cell fixation. The cells were rinsed twice with PBS and 500 μl of RNase was added to the pellets after supernatant removal. The cells were then incubated at room temperature for 20 min. Subsequently, 500 μl of propidium iodide solution was added, the mixture was poured into a cell strainer, and the cell cycle was determined by flow cytometry using an Epics XL MCL (Beckman Coulter, Inc., Fullerton, CA, USA).

## Results

**Figure 1:**
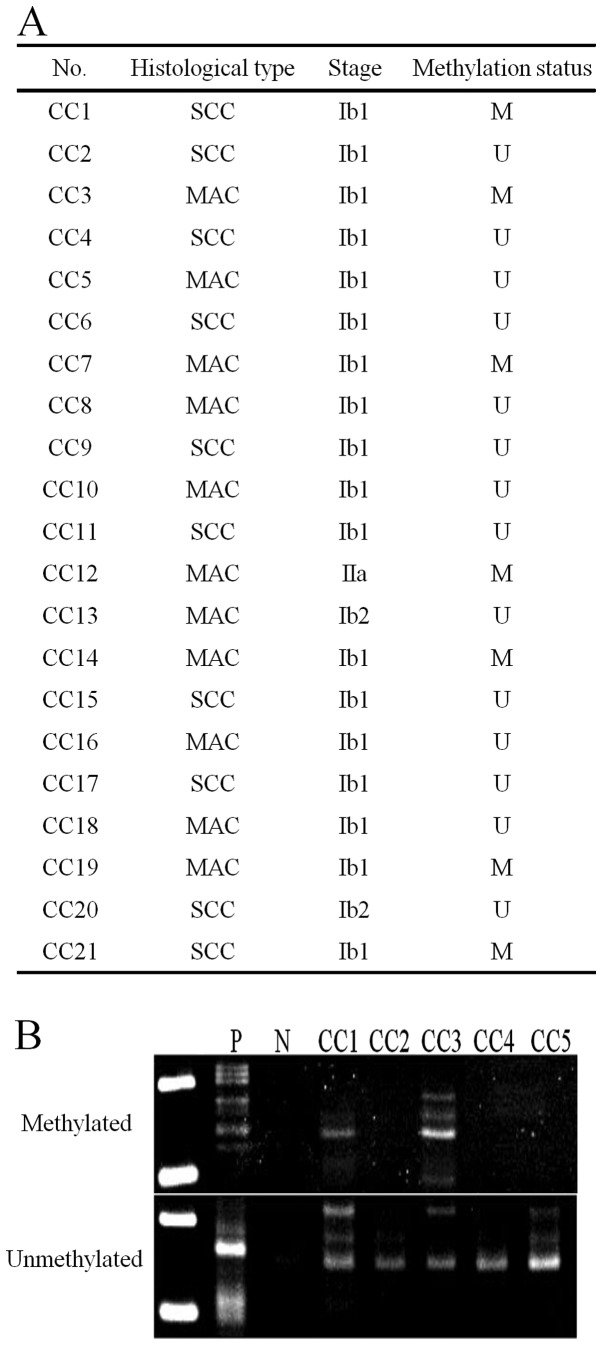
Analysis of aberrant DNA hypermethylation of the *WRN* gene in cervical cancer cytologic specimens. (A) Cervical cancer specimens and methylation status of *WRN*. CC, cervical cancer; SCC, squamous cell carcinoma; MAD, mucinous adenocarcinoma; M, methylated; U, unmethylated. (B) MSP analysis of *WRN* in cervical cancer specimens. P, positive control; N, negative control.

**Figure 2:**
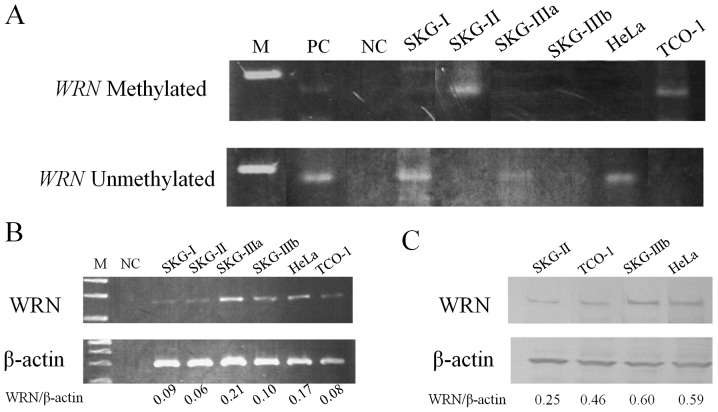
(A) MSP analysis of the *WRN* gene in cervical cancer-derived cell lines. Aberrant DNA hypermethylation of *WRN* was observed in SKG-II and TCO-1 cells. (B) Analysis of *WRN* expression in cervical cancer-derived cell lines using RT-PCR. *WRN* expression was decreased in SKG-II and TCO-1 cells, which had aberrant DNA hypermethylation of the *WRN* gene. (C) Western blot analysis of WRN in cervical cancer-derived cell lines. The WRN protein level was decreased in SKG-II and TCO-1 cells, which had aberrant DNA hypermethylation of the *WRN* gene.

Aberrant methylation of the *WRN* gene in cervical cancer was examined using specimens collected for cytology (Fig. 1). Aberrant methylation was detected in 7 (33.3%) of 21 patients, including in 2 (20%) of 10 cases of squamous cell carcinoma and 5 (45.5%) of 11 cases of adenocarcinoma. Among 6 cervical cancer-derived cell lines, aberrant methylation was detected in 2 cell lines, SKG-II and TCO-I (Fig. 2A), and mRNA and protein levels for *WRN* were lower in these cells (Fig. 2B and C).

**Figure 3:**
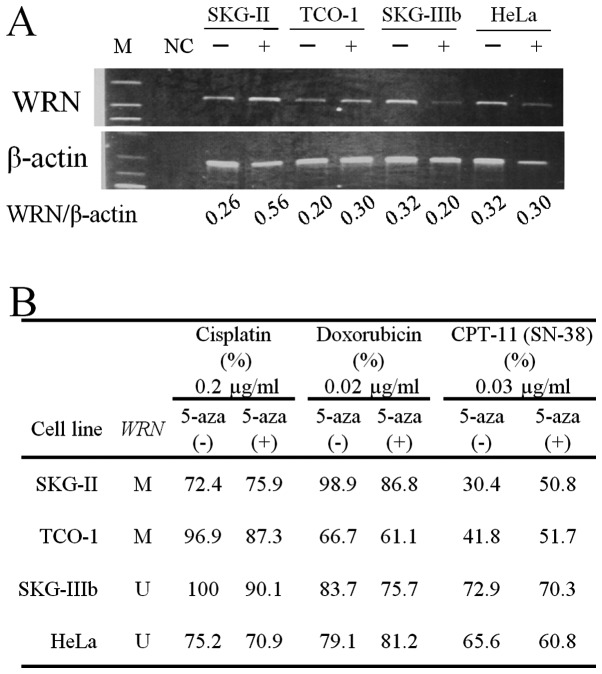
(A) Demethylation analysis of the *WRN* gene in cervical cancer-derived cell lines using RT-PCR. Treatment with a demethylating agent (5-aza) reactivated *WRN* expression in SKG-II and TCO-1 cells, which had aberrant DNA hypermethylation of the *WRN* gene. (B) Changes in sensitivity (T/C ratio) of cervical cancer-derived cell lines to anticancer agents after treatment with 5-aza. M, methylated; U, unmethylated.

Changes in WRN mRNA levels in cervical cancer-derived cell lines were analyzed before and after treatment with 5-aza, a demethylating agent (Fig. 3A). After administration of 5-aza, *WRN* mRNA increased in SKG-II and TCO-I cells, in which aberrant methylation of *WRN* was found. Sensitivity to anticancer drugs before and after treatment with 5-aza was analyzed by CD-DST, based on the T/C ratio (Fig. 3B). Sensitivity to CDDP and ADM did not change in 4 cell lines after administration of 5-aza. For CPT-11, the T/C ratio increased to >50% in SKG-II and TCO-I cells after administration of 5-aza, showing decreased sensitivity to CPT-11.

**Figure 4:**
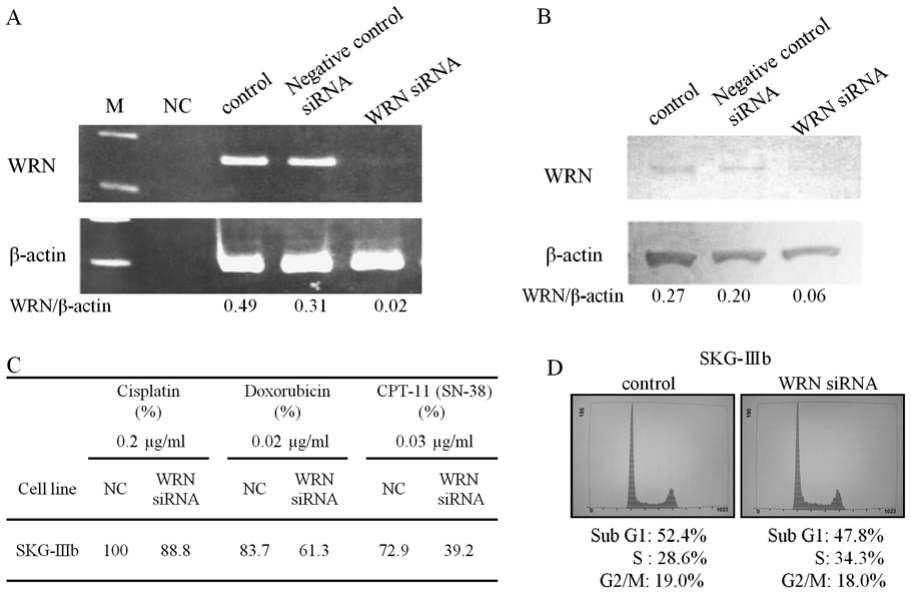
(A) RT-PCR after *WRN* knockdown by siRNA in SKG-IIIb cells. (B) Western blot analysis after *WRN* knockdown by siRNA in SKG-IIIb cells. (C) siRNA-induced changes in sensitivity (T/C ratio) of SKG-IIIb cells to anticancer agents. After suppression of *WRN* expression, only sensitivity to CPT-11 was increased. (D) Cell cycle analysis of SKG-IIIb cells using flow cytometry. After suppression of *WRN* expression, the percentage of cells in the S-phase increased.

Introduction of siRNA for *WRN* in SKG-IIIb cells decreased the levels of WRN mRNA and protein (Fig. 4A and B). The sensitivity of the cells to CPT-11 was increased by siRNA treatment based on the marked decrease in the T/C ratio in the CD-DST (Fig. 4C). Flow cytometry indicated that the percentage of S-phase cells increased from 28.6 to 34.3% after siRNA for *WRN* was introduced into SKG-IIIb cells (Fig. 4D).

## Discussion

This study provided the first evidence of a relationship of *WRN* promoter methylation with cervical cancer, with aberrant methylation of *WRN* detected in 33.3% of specimens of cervical cancer and in two cervical cancer-derived cell lines. Decreased *WRN* mRNA and protein levels were also found in both cell lines. These results suggested that aberrant methylation of *WRN* plays an important role in cervical cancer. The sensitivity to CPT-11 of cervical cancer cells with aberrant methylation of *WRN* was decreased by treatment with a demethylating agent. This treatment also increased the level of *WRN* mRNA, consistent with the general effect of demethylating agents on expression of many genes.

Selective downregulation of *WRN* expression using siRNA increased the sensitivity of cervical cancer cells to CPT-11, but not to other anticancer agents. Several previous studies have shown that *WRN* inactivation increases the anticancer effect of CPT-11 (13,21). CPT-11 acts on the covalent complex of topoisomerase I (Top-I) and DNA, and inhibits DNA replication and causes strand breaks (29–31). CPT-11 acts in S-phase and causes activation of S-phase checkpoint function via the ATR (ataxia and Rad-related protein)-CHK1 (checkpoint kinase-1) pathway (32–34). *WRN* is involved in the ATR-CHK1 pathway by recognizing the Top-I-DNA complex and detecting replication-derived DNA structures or unresolved positive supercoils (35,36). Thus, if ATR, CHK1 and *WRN* have reduced activity, cells are hypersensitive to CPT-11, and administration of CPT-11 to inactivated WS cells increases S-phase DSBs and unresolved recombination structures. A similar effect does not occur with etoposide, a topoisomerase II inhibitor (37). The S-phase checkpoint is activated upon DNA damage and is regulated by ataxia telangiectasia mutated (ATM) and ATM and Rad3-related protein (ATR) kinases. WRN is related to both kinases (38,39). Cells with inactivated *WRN* proceed to S-phase earlier than wild-type cells; however, the slow progress in late S-phase for cells with inactivated *WRN* causes the final S-phase lengths to be equal (40). In this study, siRNA for *WRN* produced a small, but insignificant, increase in the number of S-phase cells.

In chemotherapy for cervical cancer, the response rate to cisplatin is 20–30% (24) and that to monotherapy with CPT-11 has been found to be 24% (25). A combination of cisplatin and CPT-11 has a good response rate of 59% and is effective therapy (41). Taxanes also play an important role in chemotherapy for cervical cancer, with response rates of 17% for taxane monotherapy (42) and 46% for cisplatin and paclitaxel (TP) combination therapy for recurrent or advanced cervical cancer, making this regimen the current standard of care (43). We previously showed a relationship between aberrant methylation of *CHFR* and sensitivity to taxanes, and suggested that aberrant methylation of *CHFR* could serve as a molecular marker for the sensitivity of cervical cancer to anticancer drugs (44). Thus, examination of aberrant methylation of *CHFR* and *WRN* in specimens collected for cytology may be useful for prediction of treatment outcome before administration of anticancer drugs.

The standard treatment for cervical cancer is concurrent chemoradiotherapy from stages IB bulky to IIB (45). Neoadjuvant chemotherapy (NAC) is a promising approach for reduction of tumor size to increase the number of patients indicated for surgery and reduce distant metastasis through an effect on micrometastasis (46). It has been suggested that NAC could be used in stages IB2 to IIIB (47), but the efficacy of NAC remains inconclusive (48). A disadvantage of NAC is that the tumor may grow prior to the main therapy in cases that are non-responsive to NAC. However, this concern may be avoided by choice of the most effective chemotherapy based on evaluation of methylation of *CHFR* and *WRN* in specimens collected before initiation of NAC. This approach may represent a new therapeutic strategy for cervical cancer.

In conclusion, our data suggest that aberrant methylation of *WRN* plays an important role in carcinogenesis and sensitivity to CPT-11 of cervical cancer. This is the first report to show a relationship between the methylation of the *WRN* gene and sensitivity to CPT-11 in gynecologic cancer.
